# Supplementary material for: Synthesizing artificial devices that redirect cellular information at will
Source: eLife. 2018 Jan 10;7:e31936. doi: 10.7554/eLife.31936 (PMC5788502; doi:10.7554/eLife.31936)
Supplement: Supplementary file 1. — Each of these sequences consists of a complementary sequence, two copies of theophylline aptamers, and a linker sequence. [file elife-31936-supp1.docx]

**Supplementary File 1. cDNA sequences of the theophylline-induced signal-connectors targeting and suppressing Renilla luciferase mRNA translation.** Each of these sequences consists of a complementary sequence, two copies of theophylline aptamers, and a linker sequence.

| Names | Sequences |
| --- | --- |
| R1 | GCAAAAGCCTAGGCCTCCAAGGTGATACCAGCATCGTCTTGATGCCCTTGGCAGCACCCAACAACAACAACAAGGTGATACCAGCATCGTCTTGATGCCCTTGGCAGCACC |
| R2 | GTGCCTCACGACCAACTTCTGGTGATACCAGCATCGTCTTGATGCCCTTGGCAGCACCCAACAACAACAACAAGGTGATACCAGCATCGTCTTGATGCCCTTGGCAGCACC |
| R3 | GTACTCTAGCCTTAAGAGCTGGTGATACCAGCATCGTCTTGATGCCCTTGGCAGCACCCAACAACAACAACAAGGTGATACCAGCATCGTCTTGATGCCCTTGGCAGCACC |
| R4 | GTTTGCGTTGCTCGGGGTCGGGTGATACCAGCATCGTCTTGATGCCCTTGGCAGCACCCAACAACAACAACAAGGTGATACCAGCATCGTCTTGATGCCCTTGGCAGCACC |
| R5 | GGGATGATGCATCTAGCCACGGTGATACCAGCATCGTCTTGATGCCCTTGGCAGCACCCAACAACAACAACAAGGTGATACCAGCATCGTCTTGATGCCCTTGGCAGCACC |
| R6 | GGACTTACCCATTCCGATCAGGTGATACCAGCATCGTCTTGATGCCCTTGGCAGCACCCAACAACAACAACAAGGTGATACCAGCATCGTCTTGATGCCCTTGGCAGCACC |
| R7 | GTAGTGAAAGGCCAGACAAGGGTGATACCAGCATCGTCTTGATGCCCTTGGCAGCACCCAACAACAACAACAAGGTGATACCAGCATCGTCTTGATGCCCTTGGCAGCACC |
| R8 | GATGTCAGGCCACTCGTCCCGGTGATACCAGCATCGTCTTGATGCCCTTGGCAGCACCCAACAACAACAACAAGGTGATACCAGCATCGTCTTGATGCCCTTGGCAGCACC |
| R9 | GGCTCCAGTTTCCGCATGATGGTGATACCAGCATCGTCTTGATGCCCTTGGCAGCACCCAACAACAACAACAAGGTGATACCAGCATCGTCTTGATGCCCTTGGCAGCACC |
| R10 | GCCTCCCTTAACGAGAGGGAGGTGATACCAGCATCGTCTTGATGCCCTTGGCAGCACCCAACAACAACAACAAGGTGATACCAGCATCGTCTTGATGCCCTTGGCAGCACC |
| R11 | GTGTTAGGGAACTTCTTAGCGGTGATACCAGCATCGTCTTGATGCCCTTGGCAGCACCCAACAACAACAACAAGGTGATACCAGCATCGTCTTGATGCCCTTGGCAGCACC |
| R12 | GATGTACTTACCCATTTCATGGTGATACCAGCATCGTCTTGATGCCCTTGGCAGCACCCAACAACAACAACAAGGTGATACCAGCATCGTCTTGATGCCCTTGGCAGCACC |
