# Supplementary material for: Synthesizing artificial devices that redirect cellular information at will
Source: eLife. 2018 Jan 10;7:e31936. doi: 10.7554/eLife.31936 (PMC5788502; doi:10.7554/eLife.31936)
Supplement: Supplementary file 2. — Each of these sequences consists of a complementary sequence, two copies of tetracycline aptamers, and a linker sequence. [file elife-31936-supp2.docx]

**Supplementary File 2. cDNA sequences of the tetracycline-induced signal-connectors targeting and suppressing Renilla luciferase mRNA translation.** Each of these sequences consists of a complementary sequence, two copies of tetracycline aptamers, and a linker sequence.

| Names | Sequences |
| --- | --- |
| R13 | GCCTCCTCACTACTTCTGGAGGCCTAAAACATACCAGATCGCCACCCGCGCTTTAATCTGGAGAGGTGAAGAATACGACCACCTAGGCCCAACAACAACAACAAGGCCTAAAACATACCAGATCGCCACCCGCGCTTTAATCTGGAGAGGTGAAGAATACGACCACCTAGGCC |
| R14 | GGAGGCCTAGGCTTTTGCAAGGCCTAAAACATACCAGATCGCCACCCGCGCTTTAATCTGGAGAGGTGAAGAATACGACCACCTAGGCCCAACAACAACAACAAGGCCTAAAACATACCAGATCGCCACCCGCGCTTTAATCTGGAGAGGTGAAGAATACGACCACCTAGGCC |
| R15 | GGCCTTGATCTTGTCTTGGTGGCCTAAAACATACCAGATCGCCACCCGCGCTTTAATCTGGAGAGGTGAAGAATACGACCACCTAGGCCCAACAACAACAACAAGGCCTAAAACATACCAGATCGCCACCCGCGCTTTAATCTGGAGAGGTGAAGAATACGACCACCTAGGCC |
| R16 | GCGAGGCCAGGAGAGGGTAGGGCCTAAAACATACCAGATCGCCACCCGCGCTTTAATCTGGAGAGGTGAAGAATACGACCACCTAGGCCCAACAACAACAACAAGGCCTAAAACATACCAGATCGCCACCCGCGCTTTAATCTGGAGAGGTGAAGAATACGACCACCTAGGCC |
