# Supplementary material for: Synthesizing artificial devices that redirect cellular information at will
Source: eLife. 2018 Jan 10;7:e31936. doi: 10.7554/eLife.31936 (PMC5788502; doi:10.7554/eLife.31936)
Supplement: Supplementary file 3. — Each of these sequences consists of a complementary sequence, two copies of tetracycline aptamers, and a linker sequence. [file elife-31936-supp3.docx]

**Supplementary File 3.** cDNA sequences of the tetracycline-induced signal-connectors targeting and suppressing VEGF mRNA translation. Each of these sequences consists of a complementary sequence, two copies of tetracycline aptamers, and a linker sequence.

| Names | Sequences |
| --- | --- |
| R17 | GTTTCGGAGGCCCGACCGGGGGCCTAAAACATACCAGATCGCCACCCGCGCTTTAATCTGGAGAGGTGAAGAATACGACCACCTAGGCCCAACAACAACAACAAGGCCTAAAACATACCAGATCGCCACCCGCGCTTTAATCTGGAGAGGTGAAGAATACGACCACCTAGGCC |
| R18 | GCAAGGCGAGGCTCCAATGCGGCCTAAAACATACCAGATCGCCACCCGCGCTTTAATCTGGAGAGGTGAAGAATACGACCACCTAGGCCCAACAACAACAACAAGGCCTAAAACATACCAGATCGCCACCCGCGCTTTAATCTGGAGAGGTGAAGAATACGACCACCTAGGCC |
| R19 | GATCTCATCAGGGTACTCCTGGCCTAAAACATACCAGATCGCCACCCGCGCTTTAATCTGGAGAGGTGAAGAATACGACCACCTAGGCCCAACAACAACAACAAGGCCTAAAACATACCAGATCGCCACCCGCGCTTTAATCTGGAGAGGTGAAGAATACGACCACCTAGGCC |
| R20 | GCATGGTGATGTTGGACTCCGGCCTAAAACATACCAGATCGCCACCCGCGCTTTAATCTGGAGAGGTGAAGAATACGACCACCTAGGCCCAACAACAACAACAAGGCCTAAAACATACCAGATCGCCACCCGCGCTTTAATCTGGAGAGGTGAAGAATACGACCACCTAGGCC |
