# Supplementary material for: Synthesizing artificial devices that redirect cellular information at will
Source: eLife. 2018 Jan 10;7:e31936. doi: 10.7554/eLife.31936 (PMC5788502; doi:10.7554/eLife.31936)
Supplement: Supplementary file 4. — The sequence consists of a complementary sequence, two copies of eIF4G aptamers, and a linker sequence. [file elife-31936-supp4.docx]

**Supplementary File 4.** The cDNA sequence of the signal-connector targeting and enhancing Renilla luciferase mRNA translation. The sequence consists of a complementary sequence, two copies of eIF4G aptamers, and a linker sequence.

| Names | Sequences |
| --- | --- |
| R21 | GCAAAAGCCTAGGCCTCCAAGGGACACAATGGACGTCCGTAGAAACGCGTTAAGGTGAAAGTTTGAGGGCTCCTCATAACGGCCGACATGAGAGCAACAACAACAACAAGGGACACAATGGACGTCCGTAGAAACGCGTTAAGGTGAAAGTTTGAGGGCTCCTCATAACGGCCGACATGAGAG |
