# Supplementary material for: Synthesizing artificial devices that redirect cellular information at will
Source: eLife. 2018 Jan 10;7:e31936. doi: 10.7554/eLife.31936 (PMC5788502; doi:10.7554/eLife.31936)
Supplement: Supplementary file 5. — Each of these sequences consists of a complementary sequence, one copy of theophylline riboswitch, two copies of eIF4G aptamers and two linker sequences. [file elife-31936-supp5.docx]

**Supplementary File 5. cDNA sequences of theophylline-induced signal-connectors targeting and enhancing Renilla luciferase mRNA translation.** Each of these sequences consists of a complementary sequence, one copy of theophylline riboswitch, two copies of eIF4G aptamers and two linker sequences.

| Names | Sequences |
| --- | --- |
| R22 | GCAAAAGCCTAGGCCTCCAACCTCGGCCTAGGCTTTTGCGATACCAGCATCGTCTTGATGCCCTTGGCAGCGCAAAAGCCCAACAACAACAACAAGGGACACAATGGACGTCCGTAGAAACGCGTTAAGGTGAAAGTTTGAGGGCTCCTCATAACGGCCGACATGAGAGCAACAACAACAACAAGGGACACAATGGACGTCCGTAGAAACGCGTTAAGGTGAAAGTTTGAGGGCTCCTCATAACGGCCGACATGAGAG |
| R23 | GTGCCTCACGACCAACTTCTCCTCTTGGTCGTGAGGCACGATACCAGCATCGTCTTGATGCCCTTGGCAGCGTGCCTCACCAACAACAACAACAAGGGACACAATGGACGTCCGTAGAAACGCGTTAAGGTGAAAGTTTGAGGGCTCCTCATAACGGCCGACATGAGAGCAACAACAACAACAAGGGACACAATGGACGTCCGTAGAAACGCGTTAAGGTGAAAGTTTGAGGGCTCCTCATAACGGCCGACATGAGAG |
| R24 | GGAGGCCTAGGCTTTTGCAACCTCAAAGCCTAGGCCTCCGATACCAGCATCGTCTTGATGCCCTTGGCAGCGGAGGCCTACAACAACAACAACAAGGGACACAATGGACGTCCGTAGAAACGCGTTAAGGTGAAAGTTTGAGGGCTCCTCATAACGGCCGACATGAGAGCAACAACAACAACAAGGGACACAATGGACGTCCGTAGAAACGCGTTAAGGTGAAAGTTTGAGGGCTCCTCATAACGGCCGACATGAGAG |
| R25 | GTAATTGAACTGGGAGTGGACCTCTCCCAGTTCAATTACGATACCAGCATCGTCTTGATGCCCTTGGCAGCGTAATTGAACAACAACAACAACAAGGGACACAATGGACGTCCGTAGAAACGCGTTAAGGTGAAAGTTTGAGGGCTCCTCATAACGGCCGACATGAGAGCAACAACAACAACAAGGGACACAATGGACGTCCGTAGAAACGCGTTAAGGTGAAAGTTTGAGGGCTCCTCATAACGGCCGACATGAGAG |
