# Supplementary material for: Synthesizing artificial devices that redirect cellular information at will
Source: eLife. 2018 Jan 10;7:e31936. doi: 10.7554/eLife.31936 (PMC5788502; doi:10.7554/eLife.31936)
Supplement: Supplementary file 6. — Each of these sequences consists of a complementary sequence, one copy of tetracycline riboswitch, two copies of eIF4G aptamers and two linker sequences. [file elife-31936-supp6.docx]

**Supplementary File 6. cDNA sequences of tetracycline-induced signal-connectors targeting and enhancing Renilla luciferase mRNA translation.** Each of these sequences consists of a complementary sequence, one copy of tetracycline riboswitch, two copies of eIF4G aptamers and two linker sequences.

| Names | Sequences |
| --- | --- |
| R26 | GCCTCCTCACTACTTCTGGACCTCAAGTAGTGAGGAGGCAAACATACCAGATCGCCACCCGCGCTTTAATCTGGAGAGGTGAAGAATACGACCACCGCCTCCTCACAACAACAACAACAAGGGACACAATGGACGTCCGTAGAAACGCGTTAAGGTGAAAGTTTGAGGGCTCCTCATAACGGCCGACATGAGAGCAACAACAACAACAAGGGACACAATGGACGTCCGTAGAAACGCGTTAAGGTGAAAGTTTGAGGGCTCCTCATAACGGCCGACATGAGAG |
| R27 | GGAGGCCTAGGCTTTTGCAACCTCAAGCCATGGCCTCCAAACATACCAGATCGCCACCCGCGCTTTAATCTGGAGAGGTGAAGAATACGACCACCGGAGGCCTACAACAACAACAACAAGGGACACAATGGACGTCCGTAGAAACGCGTTAAGGTGAAAGTTTGAGGGCTCCTCATAACGGCCGACATGAGAGCAACAACAACAACAAGGGACACAATGGACGTCCGTAGAAACGCGTTAAGGTGAAAGTTTGAGGGCTCCTCATAACGGCCGACATGAGAG |
| R28 | GTAATTGAACTGGGAGTGGACCTCTCCCAGTTCAATTACAAACATACCAGATCGCCACCCGCGCTTTAATCTGGAGAGGTGAAGAATACGACCACCGTAATTGAACAACAACAACAACAAGGGACACAATGGACGTCCGTAGAAACGCGTTAAGGTGAAAGTTTGAGGGCTCCTCATAACGGCCGACATGAGAGCAACAACAACAACAAGGGACACAATGGACGTCCGTAGAAACGCGTTAAGGTGAAAGTTTGAGGGCTCCTCATAACGGCCGACATGAGAG |
| R29 | GTACTCTAGCCTTAAGAGCTCCTCTTAAGGCTAGAGTACAAACATACCAGATCGCCACCCGCGCTTTAATCTGGAGAGGTGAAGAATACGACCACCGTACTCTAGCAACAACAACAACAAGGGACACAATGGACGTCCGTAGAAACGCGTTAAGGTGAAAGTTTGAGGGCTCCTCATAACGGCCGACATGAGAGCAACAACAACAACAAGGGACACAATGGACGTCCGTAGAAACGCGTTAAGGTGAAAGTTTGAGGGCTCCTCATAACGGCCGACATGAGAG |
