# Supplementary material for: Synthesizing artificial devices that redirect cellular information at will
Source: eLife. 2018 Jan 10;7:e31936. doi: 10.7554/eLife.31936 (PMC5788502; doi:10.7554/eLife.31936)
Supplement: Supplementary file 10. — The sequence consists of a complementary sequence, one copy of OPN riboswitch, two copies of eIF4G aptamers and two linker sequences. [file elife-31936-supp10.docx]

**Supplementary File 10. The cDNA sequence of OPN-induced signal-connector targeting and enhancing VEGF mRNA translation.** The sequence consists of a complementary sequence, one copy of OPN riboswitch, two copies of eIF4G aptamers and two linker sequences.

| Names | Sequences |
| --- | --- |
| R33 | GTCTGGTCCCGACGATGCTGCCTCTCGTCGGGACCAGACGAATCAGTGAATGCTTATACATCGTCTGGTCCCAACAACAACAACAAGGGACACAATGGACGTCCGTAGAAACGCGTTAAGGTGAAAGTTTGAGGGCTCCTCATAACGGCCGACATGAGACAACAACAACAACAAGGGGACACAATGGACGTCCGTAGAAACGCGTTAAGGTGAAAGTTTGAGGGCTCCTCATAACGGCCGACATGAGAG |
