# Supplementary material for: Synthesizing artificial devices that redirect cellular information at will
Source: eLife. 2018 Jan 10;7:e31936. doi: 10.7554/eLife.31936 (PMC5788502; doi:10.7554/eLife.31936)
Supplement: Supplementary file 11. — The sequence consists of a complementary sequence, two copies of VEGF aptamers and one linker sequence. [file elife-31936-supp11.docx]

**Supplementary File 11. The cDNA sequence of VEGF-induced signal-connector targeting and suppressing OPN mRNA translation.** The sequence consists of a complementary sequence, two copies of VEGF aptamers and one linker sequence.

| Names | Sequences |
| --- | --- |
| R34 | GTCTGGTCCCGACGATGCTGCGGAATCAGTGAATGCTTATACATCCGCAACAACAACAACAACGGAATCAGTGAATGCTTATACATCCG |
