# Supplementary material for: Synthesizing artificial devices that redirect cellular information at will
Source: eLife. 2018 Jan 10;7:e31936. doi: 10.7554/eLife.31936 (PMC5788502; doi:10.7554/eLife.31936)
Supplement: Supplementary file 12. — The sequence consists of a complementary sequence, two copies of theophylline aptamers and one linker sequence. [file elife-31936-supp12.docx]

**Supplementary File 12. The cDNA sequence of theophylline-induced signal-connector targeting and suppressing c-Myc mRNA translation.** The sequence consists of a complementary sequence, two copies of theophylline aptamers and one linker sequence.

| Names | Sequences |
| --- | --- |
| R35 | GCAAGGAGAGCCTTTCAGAGGGTGATACCAGCATCGTCTTGATGCCCTTGGCAGCACCCAACAACAACAACAAGGTGATACCAGCATCGTCTTGATGCCCTTGGCAGCACC |
